# Healthy Lifestyle and Leukocyte Telomere Length in U.S. Women

**DOI:** 10.1371/journal.pone.0038374

**Published:** 2012-05-31

**Authors:** Qi Sun, Ling Shi, Jennifer Prescott, Stephanie E. Chiuve, Frank B. Hu, Immaculata De Vivo, Meir J. Stampfer, Paul W. Franks, JoAnn E. Manson, Kathryn M. Rexrode

**Affiliations:** 1 Department of Nutrition, Harvard School of Public Health, Boston, Massachusetts, United States of America; 2 Channing Laboratory, Department of Medicine, Brigham and Women's Hospital and Harvard Medical School, Boston, Massachusetts, United States of America; 3 College of Nursing and Health Sciences, University of Massachusetts-Boston, Boston, Massachusetts, United States of America; 4 Department of Epidemiology, Harvard School of Public Health, Boston, Massachusetts, United States of America; 5 Program in Molecular and Genetic Epidemiology, Harvard School of Public Health, Boston, Massachusetts, United States of America; 6 Department of Clinical Sciences, Genetic and Molecular Epidemiology Unit, Lund University, Malmö, Sweden; 7 Division of Preventive Medicine, Department of Medicine, Brigham and Women's Hospital and Harvard Medical School, Boston, Massachusetts, United States of America; Innsbruck Medical University, Austria

## Abstract

**Context:**

Whether a healthy lifestyle may be associated with longer telomere length is largely unknown.

**Objectives:**

To examine healthy lifestyle practices, which are primary prevention measures against major age-related chronic diseases, in relation to leukocyte telomere length.

**Design and Setting:**

Cross-sectional analysis in the Nurses' Health Study (NHS).

**Participants:**

The population consisted of 5,862 women who participated in multiple prospective case-control studies within the NHS cohort. *Z* scores of leukocyte telomere length were derived within each case-control study. Based on prior work, we defined low-risk or healthy categories for five major modifiable factors assessed in 1988 or 1990: non-current smoking, maintaining a healthy body weight (body mass index in 18.5–24.9 kg/m^2^), engaging in regular moderate or vigorous physical activities (≥150 minutes/week), drinking alcohol in moderation (1 drink/week to <2 drinks/day), and eating a healthy diet (Alternate Healthy Eating Index score in top 50%). We calculated difference (%) of the *z* scores contrasting low-risk groups with reference groups to evaluate the association of interest.

**Results:**

Although none of the individual low-risk factors was significantly associated with larger leukocyte telomere length *z* scores, we observed a significant, positive relationship between the number of low-risk factors and the *z* scores. In comparison with women who had zero low-risk factors (1.9% of the total population) and were, therefore, considered the least healthy group, the leukocyte telomere length *z* scores were 16.4%, 22.1%, 28.7%, 22.6%, and 31.2% (P for trend = 0.015) higher for women who had 1 to 5 low-risk factors, respectively.

**Conclusions:**

Adherence to a healthy lifestyle, defined by major modifiable risk factors, was associated with longer telomere length in leukocytes.

## Introduction

The risk of developing many major chronic diseases, including cardiovascular disease (CVD), cancer, and type 2 diabetes, rises substantially with age. However, the risk of developing these diseases varies considerably for individuals of the same age, suggesting the hypothesis that “biological aging” rather than “chronological aging” may be the more important component of the increasing risks across the life span. Accumulating evidence suggests that the attrition of telomeres, special chromatin structures that cap the ends of eukaryotic chromosomes and maintain genome stability [1], [2], may reflect biological aging. At the cellular level, progressive loss of telomeric DNA eventually leads to cell death or senescence [3], [4], [5], and may subsequently contribute to the increased risk of developing age-related chronic conditions [6], [7], [8], [9].

Interestingly, at the population level, studies have suggested that the telomere attrition is likely a modifiable factor as there is substantial variability in the rate of telomere shortening that is independent of chronological age. Such telomere length variability may be partially explained by lifestyle practices [10]. Meanwhile, a growing literature has consistently shown that a substantial proportion of chronic diseases, including coronary heart disease, stroke, diabetes, as well as early mortality, may be attributable to failing to adopt a composite healthy lifestyle that combines multiple low-risk factors, including non-smoking, regular physical activity, a healthy diet, maintaining a healthy body weight, and light-to-moderate alcohol consumption [11], [12], [13], [14], [15], [16], [17], [18]. More importantly, all of these studies have demonstrated that the joint effects of multiple low-risk factors are much more pronounced than the effect of any single factor. However, few studies [19] have been conducted to examine the association of a combination of low-risk lifestyle factors with telomere length, which may potentially explain the protective effects of healthy lifestyle on age-related health conditions.

We, therefore, took advantage of the rich data collected in the Nurses' Health Study (NHS) cohort to explicitly evaluate combinations of low-risk, healthy lifestyle practices in relation to leukocyte telomere length (LTL) in women at midlife, a life-stage at considerable risk of developing chronic diseases [20], [21], [22].

## Methods

### Study Population

The NHS was established in 1976 when 121,700 female registered nurses aged 30–55 years were enrolled through a questionnaire inquiring about history of chronic diseases and demographic and lifestyle characteristics. Since baseline, follow-up questionnaires had been administered every two years to update the information of disease incidence, lifestyle practices, and clinical risk factors. In the NHS, self-reports of major chronic diseases (such as cancer, diabetes, coronary heart disease, and stroke) were confirmed by study physicians through medical record review, pathology report review, telephone interview, or supplementary questionnaire inquiries [23], [24], [25]. Deaths were identified by reports from next of kin, postal authorities, or by a search of the National Death Index. At least 98% of deaths among the NHS participants were identified [26]. Through 2002, follow-up rates have exceeded 95%.

In 1989–1990, 32,826 participants provided blood samples [27], which were immediately processed upon arrival and aliquoted into cryotubes as plasma, buffy coat, and red blood cells. Among this cohort, multiple prospective nested case-control studies of cancers and CVD were conducted [28], [29], [30]. All of these case-control studies used the same design: for each incident case of a disease, a risk-set sampling scheme was used to select one to three controls from the remaining participants in the blood sub-cohort who were free of that disease when the case was diagnosed. All cases and controls were free of the specific disease of interest in each individual study when blood was drawn. In addition, cases and controls were matched for menopausal status and postmenopausal hormone use (except for the myocardial infarction case-control study), age, and time of blood draw, as well as other factors that were carefully chosen for each individual study. In addition to these case-control studies, telomere length data were also available in a study of cognitive function [31]. In total, the current analysis included data of 7,116 participants from nine studies (myocardial infarction, stroke, cognitive function, breast cancer, endometrial cancer, pancreatic cancer, and three skin cancer studies), in which LTL was measured. In published data based on some of these studies, shorter telomere length was not significantly associated with cancer risks [28], [29], [30], but weakly correlated with more cognitive decline [31].

The study protocol was approved by the institutional review board of the Brigham and Women's Hospital and the Human Subjects Committee Review Board of Harvard School of Public Health. Written informed consent was obtained from all participants.

### Measurement of Leukocyte Telomere Length

All buffy-coat cryotubes were stored in the vapor phase of liquid nitrogen freezers at ≤−130°C. Genomic DNA was extracted from buffy coat fractions using the QIAmp (Qiagen, Chatsworth, CA) 96-spin blood protocol. A pico-green quantitation using a Molecular Devices 96-well spectrophotometer was used and results were confirmed by using a Nanodrop SD-1000 spectrophotometer. Subsequent standardization by drying down the genomic DNA and re-suspending ensured accurate and uniform DNA concentrations. For all 9 studies, DNA was extracted at the same lab and then stored in freezers at ≤−80°C. Of these 9 studies that contributed to the current analysis, telomere length was measured within a month after DNA was extracted for 4 studies. This time lag was within 1 year for 3 studies or 2–4 years for the rest 2 studies.

Relative average LTL was assessed by a modified high-throughput version [32] of the real-time PCR-based telomere assay developed by Cawthon et al [33]. In this assay, the ratio of telomere repeat copy number to single-copy gene copy number (T∶S ratio) was derived and the relative LTL was measured as the exponentiated T∶S ratio. All samples for both the telomere and single-copy gene (ribosomal protein, large, P0; *RPLP0*) reactions were processed in triplicate. Technicians and laboratory personnel were blinded as to the disease status of the participants. Samples were assayed by the same technicians in a random sequence under identical conditions. In each contributing study, we included 10% quality-control (QC) samples in each batch, and each QC sample was evenly split into halves. Based on the repeated measurements of these QC samples, we calculated the coefficient of variation (CV) to evaluate the performance of telomere length assays. The CVs ranged from 0.2% to 3.1% for the single-copy gene assay, 0.6% to 2.1% for the telomere assay, and 10.8 to 16.0% for the exponentiated T∶S ratio. Because telomere length was assayed during 2007–2010 for the 9 studies in various batches, in the current analysis, to minimize the impact of potential batch shift on LTL measurements across different studies, we log-transformed the LTL and then calculated a *z* score of LTL by standardizing the LTL in comparison to the mean within each individual study [34].

### Assessment of Lifestyle Practices

Beginning in 1976, the NHS has collected and updated biennially the information on a wide array of demographic and lifestyle risk factors, including detailed history of cigarette smoking, family history of chronic diseases, personal medical history, and use of aspirin. Body weight and height were self-reported with high accuracy [35]. Body mass index (BMI) was calculated as weight in kilograms divided by the square of height in meters (kg/m^2^) to estimate overall adiposity. In 1988, a validated questionnaire [36] for physical activity was embedded into the follow-up questionnaire to evaluate the average time per week in the past year spent on leisure-time physical activities including walking or hiking outdoors, bicycling, lap swimming, tennis, and calisthenics/aerobics/aerobic dance/rowing machine. For each question, there were 10 possible coding responses, ranging from “zero” to “11+ hours/week”. We further inquired about the usual walking pace of NHS participants; coding responses included easy (<2 miles/hour [MPH]), normal (2.0–2.9 MPH), brisk (3.0–3.9 MPH), and very brisk (≥4.0 MPH). Walking at a brisk or very brisk pace was considered as a moderate physical activity while jogging, running, bicycling, swimming, tennis, and aerobics activities were considered as vigorous physical activities.

In 1980, a 61-item semi-quantitative food frequency questionnaire (FFQ) was sent to NHS participants to assess their diet in the past year. In 1984, 1986, and every 4 years thereafter, an expanded FFQ containing 116–130 food items was sent to the NHS participants to update their diet. In these FFQs, we inquired about the frequency of consuming each food item of a pre-specified standard portion size. The validity of these FFQs was documented in previous studies [37]. To summarize the overall quality of diet, we created the Alternate Healthy Eating Index (AHEI), which was based on the U.S. Department of Agriculture Healthy Eating Index [38]. Briefly, the AHEI index summarized higher intakes of vegetables, fruit, nuts, soy, and cereal fiber, higher ratios of chicken plus fish to red meat and polyunsaturated to saturated fat, lower intake of *trans* fat; and multivitamin use of ≥5 years. Possible values of the AHEI index ranged from 2.5 (worst) to 77.5 (best). For each alcoholic beverage, a standard portion size (1 glass/bottle/can for beer, 4 oz. glass for wine, and 1 drink/shot for liquor) was specified and the participants were asked how often, on average, they consumed the alcoholic beverage of that specified amount. There were nine possible coding responses, ranging from “never or less than once per month” to “six or more times per day”. Frequency of alcohol consumption was derived by summing up the frequency of consumption for each individual alcoholic beverage type.

### Definition of Healthy Lifestyle Pattern

A healthy lifestyle was defined using five components including smoking, physical activity, adiposity, alcohol use, and diet. For each factor, we created a binary low-risk or healthy variable, defined as non-current smoking, 18.5 kg/m^2^≤BMI<25.0 kg/m^2^, moderate or vigorous physical activities ≥150 minutes/week [39], moderate alcohol consumption (1 drink/week to <2 drinks/day), and AHEI score in top 50%, respectively. Similar definitions of healthy lifestyle pattern were used in previous analyses in the NHS cohort [12], [13], [17]. The assessments of these variables were based on 1990 questionnaire, except physical activity which was assessed in 1988 questionnaire.

The associations between healthy lifestyles and multiple chronic diseases have been well documented in previous studies [12], [13], [14], [15], [17], [18]. For example, in comparison to men or women with no low-risk factors, those who adhered to healthy lifestyle patterns comprised of multiple low-risk lifestyle and dietary factors had 84% lower risk of coronary heart disease [13], 69% to 81% lower risk of total or ischemic strokes [17], 52% lower risk of heart failure [15], 91% lower risk of type 2 diabetes [12], and 62% to 65% lower mortality rate [14], [18].

### Exclusion Criteria

In the current analysis, we included both cases and controls to maximize our statistical power. We applied two exclusion criteria. First, to minimize confounding by existing chronic diseases on the associations of interest, we excluded 637 participants who were diagnosed with heart disease, stroke, diabetes, and cancers prior to blood draw. Second, we excluded 617 participants who had missing values for any of the 5 lifestyle or dietary variables. After these two exclusions, a total of 5,862 (82.4% of total eligible participants) women were included in the current analysis.

### Statistical Analysis

We calculated Spearman correlation coefficients of LTL *z* score with age and lifestyle factors. To estimate potential dose-response relationship between LTL *z* score and the combinations of low-risk factors, we used generalized linear regression to calculate least-square (LS) means of LTL *z* score by levels or categories of lifestyle factors. These LS means were adjusted for age at blood draw and its quadratic term, postmenopausal status (yes, no), postmenopausal hormone therapy (never, past, current), use of aspirin (<1 tablet/wk, 1–2 tablet/wk, 3–6 tablet/wk, 7–14 tablet/wk, 15+ tablet/wk), family history of myocardial infarction, diabetes, or cancer (yes, no), and the case-control status in the contributing studies to control for confounding. Since *z* scores of relative LTL may be difficult to interpret, we further calculated the relative difference (% of difference) for each category in comparison with a reference group. This analysis was conducted using a macro, *%Robreg9* (available on request), which calculates point estimates and empirical standard errors of effect on the unitless percent change scale in robust linear regression models when the dependent variable is on the log scale [40], [41]. More details for this method were introduced at the Methods S1. To estimate P for linear trend between the number of low-risk factors and telomere length *z* score, we entered an ordinal score (0 to 5, corresponding to the number of the factors) into the multivariate models.

All P values are two-sided. Ninety-five percent confidence intervals (95% CI) were calculated for ORs. Data were analyzed with the Statistical Analysis Systems software package, version 9.1 (SAS Institute, Inc., Cary, North Carolina).

## Results

### Primary Analysis

**TABLE 1** shows the baseline characteristics of the study participants in comparison with the rest of NHS cohort who did not meet the exclusion criteria set for the current analysis, as well as with those who were excluded following the exclusion criteria. Because age, postmenopausal status and hormone use were matching factors for most of the contributing case-control studies, our study participants were slightly older and more likely to use hormone therapy after menopause than the rest of NHS cohort. Otherwise, we observed largely similar distributions of lifestyle and dietary factors between these two groups after age-standardization. Distributions of family history of major diseases were slightly different between these two groups, although no consistent pattern was observed. In contrast, the excluded participants tended to have a less optimal distribution of low-risk factors. For example, excluded participants were heavier, less physically active, and more likely to be smokers and had a lower AHEI score in comparison with the other two groups, whereas all three groups had the same levels of alcohol consumption. In addition, the cases and controls who were selected for the original case-control studies had similar distribution of these factors (**TABLE S1**).

**Table 1:** Baseline characteristics* of the study participants in the current analysis in comparison with the rest of Nurses' Health Study participants, 1990.

Variables	Study participants	NHS cohort†	Excluded participants‡
N	5862	52884	50765
Age (year)	58.7±0.09	56.0±0.03	57.3±0.03
BMI (kg/m^2^)	25.2±0.3	25.5±0.3	26.3±0.3
Moderate to vigorous physical activity (minutes/week)§	135.0±11.9	126.2±11.8	111.8±11.2
Smoking status (%)			
Never smoked	43.6	44.8	41.6
Past smoker	41.3	39.2	37.2
Current smoker	15.2	16.0	21.1
Alcohol intake (drink/day)	0.4±0.04	0.4±0.04	0.4±0.04
AHEI diet score	39.6±0.5	38.6±0.5	38.1±0.5
Postmenopausal status (%)			
Premenopausal	21.5	26.5	28.0
Never used HRT	23.4	29.4	29.5
HRT past user	13.3	16.3	18.5
HRT current user	41.8	27.8	24.0
Use of aspirin (%)			
<1 tablet/wk	35.5	37.2	38.4
1–6 tablet/wk	48.7	47.1	45.5
7+ tablet/wk	15.8	15.7	16.1
Family history of myocardial infarction (%)	18.6	19.0	19.2
Family history of diabetes (%)	26.7	26.1	21.9
Family history of cancer (%)	16.4	14.0	10.8

Abbreviations: NHS, the Nurses' Health Study; BMI, body mass index; HRT, hormone replacement therapy; AHEI, alternate healthy eating index.

* Values were age-adjusted mean±SE for continuous variables or age-adjusted proportion for categorical variables, except age *per se*.

† The same exclusion criteria were applied to the rest NHS participants. Therefore, this group was primarily consisted of participants who responded to 1990 FFQ.

‡ Excluded by two reasons, i.e., missing values of low-risk factors (83.2%) due to non-response to 1988 or 1990 follow-up questionnaires and development of prevalent chronic diseases at baseline (16.8%).

§ Assessed by 1988 questionnaire. Moderate physical activities included walking at a brisk (3.0–3.9 MPH) or very brisk (≥4.0 MPH) pace. Vigorous physical activities included jogging (≥10 min/mile), running (<10 min/mile), bicycling, lap swimming, tennis, and calisthenics/aerobics/aerobic dance/rowing machine. All other variables were assessed using the 1990 questionnaire, which was administered primarily during blood collection.

We calculated Spearman correlation coefficients (*r_s_*) between LTL *z* score and age and the modifiable factors of interest. As expected, we observed a significant, inverse correlation (*r_s_* = −0.10, P<0.0001) for age. After controlling for age, we observed a weak correlation for smoking status (*r_s_* = −0.03, P = 0.03), whereas there were no correlations for BMI (*r_s_* = −0.02, P = 0.15), physical activity (*r_s_* = 0.02, P = 0.18), alcohol consumption (*r_s_* = 0.001, P = 0.93), and AHEI score (*r_s_* = 0.02, P = 0.09). Similar patterns of association were found when modeling these data categorically (**TABLE 2**). None of these individual comparisons reached statistical significance in multivariate analyses, except smoking status and AHEI score. In comparison with current smokers, women who never smoked had an 8.0% (95% CI: 0.5%, 16.7%) higher LTL *z* scores. This figure was 7.3% (95% CI: 0.0%, 15.2%) comparing the 3^rd^ AHEI quartile with the lowest quartile. After mutual adjustment for other lifestyle and dietary factors, these associations were attenuated to 7.6% (95% CI: −0.4%, 16.2%) for smoking and 6.1% (95% CI: −1.3%, 14.1%) for AHEI score. The associations for other factors remained non-significant after mutual adjustments.

**Table 2:** Multivariate-adjusted least-squares (LS) means of *z* score of leukocyte telomere length (LTL) and relative LTL difference by categories of modifiable risk factors in women.

Risk factors	n (%)	Model 1*		Model 2†	
		LS means (SE)	% Difference‡ (95% CI)	LS means (SE)	% Difference‡ (95% CI)
Smoking status					
Never smoked	2591 (44.2)	0.043 (0.020)	8.0% (0.5%, 16.7%)	0.044 (0.020)	7.6% (−0.4%, 16.2%)
Past smoker	2425 (41.4)	0.004 (0.020)	4.1% (−3.5%, 12.3%)	0.001 (0.020)	2.9% (−4.8%, 11.2%)
Current smoker	846 (14.4)	−0.036 (0.034)	Reference	−0.029 (0.035)	Reference
Body mass index (kg/m^2^)					
<18.5	72 (1.2)	−0.085 (0.117)	−5.7% (−25.1%, 18.7%)	−0.084 (0.118)	−6.2% (−25.4%, 18.1%)
18.5–24.9	3288 (56.1)	0.033 (0.017)	6.1% (−2.2%, 15.1%)	0.031 (0.018)	5.3% (−3.1%, 14.4%)
25.0–29.9	1753 (29.9)	0.005 (0.024)	3.2% (−5.4%, 12.5%)	0.006 (0.024)	2.6% (−5.9%, 12.0%)
≥30	749 (12.8)	−0.026 (0.037)	Reference	−0.021 (0.037)	Reference
Moderate to vigorous physical activity (minutes/week)					
≥150	2072 (35.4)	0.045 (0.022)	6.2% (−0.5%, 13.3%)	0.037 (0.022)	4.3% (−2.4%, 11.5%)
60–149	1000 (17.1)	0.016 (0.031)	3.1% (−4.6%, 11.4%)	0.015 (0.032)	2.0% (−5.6%, 10.2%)
<60	1069 (18.2)	0.005 (0.030)	2.0% (−5.4%, 10.0%)	0.007 (0.031)	1.2% (−6.2%, 9.1%)
0	1721 (29.4)	−0.015 (0.024)	Reference	−0.005 (0.024)	Reference
AHEI diet score¶ (range)					
Quartile 4 (46.6–73.2)	1465 (25.0)	0.036 (0.026)	6.8% (−0.6%, 14.7%)	0.027 (0.027)	5.0% (−2.6%, 13.1%)
Quartile 3 (39.9–46.6)	1467 (25.0)	0.041 (0.026)	7.3% (0.0%, 15.2%)	0.037 (0.026)	6.1% (−1.3%, 14.1%)
Quartile 2 (33.5–39.8)	1465 (25.0)	0.015 (0.026)	4.6% (−2.7%, 12.5%)	0.019 (0.026)	4.2% (−3.2%, 12.1%)
Quartile 1 (9.1–33.4)	1465 (25.0)	−0.030 (0.026)	Reference	−0.022 (0.027)	Reference
Alcohol use					
≥2 drinks/day	348 (5.9)	0.054 (0.053)	3.9% (−6.3%, 15.3%)	0.067 (0.054)	5.4% (−5.2%, 17.2%)
1 drink/week to <2 drinks/day	2744 (46.8)	0.012 (0.019)	−0.3% (−5.9%, 5.5%)	0.011 (0.019)	−0.3% (−6.0%, 5.7%)
<1 drink/week	663 (11.3)	0.012 (0.039)	−0.4% (−8.6%, 8.6%)	0.012 (0.039)	−0.2% (−8.5%, 8.7%)
Non-user	2107 (35.9)	0.015 (0.022)	Reference	0.014 (0.022)	Reference

* Model 1 was adjusted for age at blood draw and its quadratic term, postmenopausal status (yes, no), hormone replacement therapy (never, past, current), use of aspirin (<1 tablet/wk, 1–2 tablet/wk, 3–6 tablet/wk, 7–14 tablet/wk, 15+ tablet/wk), case-control status in each original studies (yes, no), and family history of myocardial infarction, diabetes, or cancer (yes, no).

† Based on model 1, these modifiable risk factors were mutually adjusted for, i.e., we mutually controlled for smoking status (never smoked, past smoker, or current smoker), body mass index (<18.5 kg/m^2^, 18.5–24.9 kg/m^2^, 25.0–29.9 kg/m^2^, or ≥30 kg/m^2^), moderate to vigorous physical activity (≥150 minutes/week, 60–149 minutes/week, <60 minutes/week, or 0 minutes/week), AHEI diet score (in quartiles), and alcohol use (≥2 drinks/day, 1 drink/week to <2 drinks/day, <1 drink/week, or non-user).

‡ % Difference measures the change of standardized LTL for each category in proportion to the standardized LTL of the reference category for each factor after multivariate adjustment.

¶ AHEI diet score summarized intakes of *trans* fat, polyunsaturated to saturated fat ratio, ratio of chicken and fish to red meat, fruits, vegetables, soy, nuts, cereal fiber, and multivitamin use.

In a separate analysis, we evaluated LTL *z* score between the optimal low-risk group and all other women for each modifiable factor. Similarly, none of the individual factors were associated with longer telomere length (**TABLE 3**). After adjustment for covariates, including mutual adjustment of the other lifestyle factors, women who were not current smokers had a 5.2% (95% CI: −2.1%, 13.1%) higher LTL *z* scores than all other women. The relative difference was 3.6% (95% CI: −1.7%, 9.2%) for BMI (between 18.5 and 24.9 kg/m^2^ vs. other), 3.4% (95% CI: −2.2%, 9.2%) for physical activity (≥150 minutes/week vs. other), 3.3% (95% CI: −2.0%, 8.9%) for AHEI score (top 50% vs. bottom 50%), and −0.9% (95% CI: −5.9%, 4.4%) for alcohol use (between 1 drink/week to <2 drinks/day vs. other).

**Table 3:** Multivariate-adjusted least-squares (LS) means of *z* score of leukocyte telomere length (LTL) and relative LTL difference by low vs. high risk groups for each modifiable risk factor in women.

Risk factors	n(%)	Model 1*		Model 2†	
		LS means (SE)	% Difference‡ (95% CI)	LS means (SE)	% Difference‡ (95% CI)
Smoking status					
Non-current smoking	5016 (85.6)	0.024 (0.014)	6.2% (−0.9%, 13.9%)	0.023 (0.014)	5.2% (−2.1%, 13.1%)
All other women	846 (14.4)	−0.036 (0.034)	Reference	−0.028 (0.035)	Reference
Body mass index					
18.5–24.9 kg/m^2^	3288 (56.1)	0.033 (0.017)	4.0% (−1.2%, 9.6%)	0.031 (0.018)	3.6% (−1.7%, 9.2%)
All other women	2574 (43.9)	−0.007 (0.020)	Reference	−0.004 (0.020)	Reference
Moderate to vigorous physical activity					
≥150 minutes/week	2072 (35.4)	0.045 (0.022)	4.7% (−0.7%, 10.5%)	0.037 (0.022)	3.4% (−2.2%, 9.2%)
All other women	3790 (64.7)	−0.001 (0.016)	Reference	0.004 (0.016)	Reference
Diet					
AHEI score¶ in top 50%	2932(50.0)	0.038 (0.018)	4.6% (−0.6%, 10.1%)	0.032 (0.019)	3.3% (−2.0%, 8.9%)
All other women	2930 (50.0)	−0.007 (0.018)	Reference	−0.001 (0.019)	Reference
Alcohol use					
1 drink/week to <2 drinks/day	2744 (46.8)	0.012 (0.019)	−0.7% (−5.7%, 4.5%)	0.011 (0.019)	−0.9% (−5.9%, 4.4%)
All other women	3118 (53.2)	0.019 (0.018)	Reference	0.020 (0.018)	Reference

* Model 1 was adjusted for the same set of covariates in model 1, Table 2.

† Based on model 1, these modifiable risk factors were mutually adjusted for, i.e., we mutually controlled for smoking status (never smoked, past smoker, or current smoker), body mass index (<18.5 kg/m^2^, 18.5–24.9 kg/m^2^, 25.0–29.9 kg/m^2^, or ≥30 kg/m^2^), moderate to vigorous physical activity (≥150 minutes/week, 60–149 minutes/week, <60 minutes/week, or 0 minutes/week), AHEI diet score (in quartiles), and alcohol use (≥2 drinks/day, 1 drink/week to <2 drinks/day, <1 drink/week, or non-user).

‡ % Difference measures the change of standardized LTL for each category in proportion to the standardized LTL of the reference category for each factor after multivariate adjustment.

¶ AHEI diet score summarized intakes of *trans* fat, polyunsaturated to saturated fat ratio, ratio of chicken and fish to red meat, fruits, vegetables, soy, nuts, cereal fiber, and multivitamin use.

**Figure 1:**
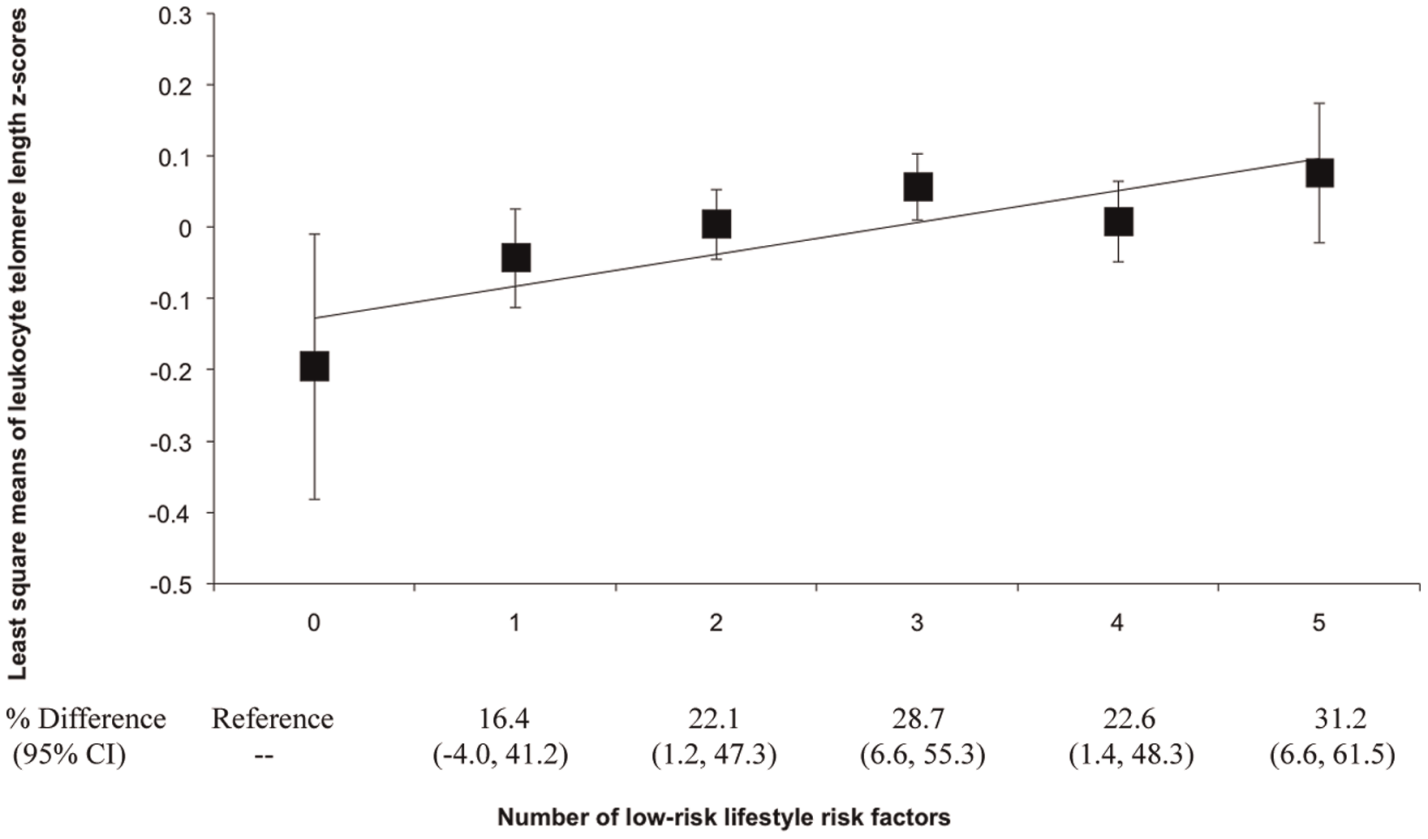
LS means of leukocyte telomere length *z* scores by numbers of low-risk lifestyle practices. Least-square means were adjusted for the same set of covariates for model 1 in Table 2. Low-risk group for each lifestyle factor was defined as non-current smoking, moderate alcohol use (1 drink/week to <2 drinks/day), a healthy body weight (18.5 kg/m^2^≤BMI<25.0 kg/m^2^), exercising at moderate to vigorous intensity (≥150 minutes/week), or AHEI diet score in top two quartiles.

Subsequently, we examined the association between the total number of low-risk factors and LTL *z* score (**FIGURE 1** and **TABLE S2**). Of 5862 participants, 1.9% had no low-risk factors, i.e., they constituted the least healthy group. The proportions of women who had only 1 to all 5 (the most healthy group) low-risk factors were 13.7%, 27.2%, 30.0%, 20.4%, and 6.8%, respectively. We observed increased LTL *z* scores with increasing number of low-risk factors. In comparison to women who had no low-risk factors, women who had 1 to 5 low-risk factors had 16.4% (95% CI: −4.0%, 41.2%), 22.1% (95% CI: 1.2%, 47.3%), 28.7% (95% CI: 6.6%, 55.3%), 22.6% (95% CI: 1.4%, 48.3%), and 31.2% (95% CI: 6.6%, 61.5%; P for trend = 0.015) higher LTL z score, respectively.

### Secondary Analysis

In a secondary analysis, we examined the robustness of these associations by excluding studies for which DNA was extracted more than 1 year before telomere length assay. When we excluded the breast cancer study that had DNA extracted 4 years before telomere assay, we observed similar associations: in comparison with participants who had no low-risk factors, the LTL z score was 12.3%, 19.2%, 28.2%, 20.2%, and 27.2% (95% CI: 1.1%, 61.3%; P for trend = 0.02) higher for women who had 1 to 5 low-risk factors. These figures were 11.0%, 17.6%, 26.5%, 19.0%, and 26.3% (95% CI: −0.3%, 59.9%; P for trend = 0.02) after melanoma study (the time lag between DNA extraction and telomere length assay was 2 years) was further excluded. Furthermore, we also examined the associations within 3,580 controls to evaluate the robustness of the results. We observed largely similar associations within the controls only, although most of these associations did not reach statistical significance likely due to substantially reduced statistical power. For example, in comparison with participants who had no low-risk factors, the LTL z score was 9.8%, 9.6%, 15.0%, 17.0%, and 28.1% (95% CI: −2.8%, 68.7%; P for trend = 0.017) higher for women who had 1 to 5 low-risk factors. In a separate analysis, we excluded alcohol consumption from the low-risk factors and repeated the analysis to be comparable with the only prior study that was conducted in men [19]. In this analysis, in comparison with women who had zero low-risk factors, women who had 1–4 low-risk factors had 21.2%, 25.1%, 31.5%, and 28.1% (95% CI: 9.6%, 49.7%; P for trend = 0.003) higher LTL *z* score, respectively. Lastly, we evaluated the correlation among the 5 low-risk factors in our study population (**TABLE S3**). Most of these pair-wise associations were highly significant, corroborating the inter-correlation among these lifestyle and dietary factors. Exceptions were only found between moderate drinking and non-smoking or healthy diet.

## Discussion

In these middle-aged U.S. women who were generally healthy at baseline, optimal lifestyle practices, defined by five low-risk, healthy lifestyle factors for chronic disease (non-current smoking, engaging in regular moderate to vigorous physical activity, maintaining an optimal body weight, eating a healthy diet, and consuming alcohol in moderation), were significantly associated with longer LTL. Although each individual low-risk factor was associated with longer LTL only weakly, the combined effect of these low-risk factors was much stronger than that of any single factor.

### Results in relation to other studies

Associations between lifestyle and dietary factors and LTL have been examined previously, but the results have been inconsistent. For example, current smoking was associated with shorter LTL in some [42], [43], [44], but not all studies [19], [45], [46], [47]. Similarly, mixed results were observed for overall or central obesity [43], [44], [45], [46], [47], [48], [49], [50], physical activity [19], [45], [46], [48], and dietary factors, including alcohol [19], [45], [46], [51], [52]. These studies varied in sample size, participants' characteristics, laboratory methods for LTL measurement, and instruments for measuring lifestyle factors. More importantly, since these lifestyle factors are typically intertwined, mutual confounding by each other may result in spurious findings. At the same time, given the correlations among these factors, it is useful to examine the overall lifestyle pattern, rather than each factor in isolation. Thus far, only one study examined the joint effects of healthy lifestyle factors on telomere length. In U.S. men a healthy lifestyle defined by four low-risk factors (low or no cigarette use, higher fruit and vegetable intake, lower BMI, and more physical activity) was significantly associated with higher LTL [19]. To our knowledge, the current analysis is the first study to evaluate this association explicitly in women, and we found consistent results indicating that the joint effects of these factors are stronger than the associations with individual factors.

### Possible mechanisms

In most human adult somatic cells, except human germline and stem cells, the activity of telomerase is absent or not sufficient to further elongate telomeric DNA sequence, leading to an average telomere attrition rate of 15–150 base pairs per cell division [53]. In addition, mounting evidence suggests that inflammation and oxidative stress, two closely related phenomena, can accelerate telomere attrition for the entire organism and multiple types of cells [54]. Telomeres are highly sensitive to the damage rendered by oxidative stress because of the G-rich nucleotide sequence [55]. Oxidative stress causes single-strand breaks in telomeres and subsequent telomere shortening, whereas high antioxidative capacity slows telomere attrition [55], [56], [57], [58]. Similarly, chronic inflammation promotes telomere shortening, especially in peripheral leukocytes, possibly by increasing cell turn-over [59]. In addition, human studies suggest that increased insulin resistance may contribute to telomere attrition [49], [59]. These mechanisms likely explain the strong protective effect of healthy lifestyle behaviors on LTL, given the established protective effects that these behaviors convey on insulin resistance, chronic inflammation, and oxidative stress [60].

### Strengths and limitations

There are several caveats to the current analyses that deserve consideration. First, we measured telomere length only in leukocytes. Whether these associations can be extrapolated to other tissues is unclear. However, studies have demonstrated robust correlations between LTL and telomere length in other tissues, including skin, synovial tissue, vascular wall, and umbilical artery [61], [62], [63]. Therefore, LTL may serve as a surrogate marker for telomere length in other tissues. Second, although the methods that were used to assess lifestyle and dietary factors have been validated and the accuracy was proven to be satisfactory in the NHS cohort, the measurements of lifestyle and dietary factors are subject to measurement errors. Similarly, our measurement of LTL had measurement error as well [64]. The use of LTL *z* score minimized between-batch variation of telomere length assay, although the same *z* score may correspond to different categories of individual lifestyle factors in each dataset depending on the dataset-specific distribution of these factors. However, since we followed strict laboratory protocol ensuring that all of these measurement errors were not correlated with each other, such measurement errors were more likely to attenuate true associations. Third, measurement error of telomere length assay may also result from DNA oxidation or degradation during long-term storage. In the NHS, buffy coat fractions were stored at ≤−130°C, and extracted DNA samples were stored at ≤−80°C before telomere length assay. Evidence has suggested that buffy coats are a reliable source of good-quality DNA for genome-wide association analysis and other genetic testing after up to 9 years' storage in a −80°C frozen state [65], although more data are needed to confirm whether this is also true for telomere length assays. Nonetheless, when we excluded two studies for which extracted DNA was stored in freezers ≤−80°C for more than 1 year, we observed similar associations. Fourth, a cross-sectional study such as this cannot determine a temporal relationship between lifestyle exposures and telomere length. However, it is unlikely that telomere length itself would have caused the participants' lifestyle behaviors to change, as the participants were unaware of their telomere status at the time when lifestyle data were obtained. Fifth, our study participants are primarily comprised of registered nurses of European ancestry. It is unknown whether our findings can be generalized to populations of other ethnicities or with different characteristics that may modify these associations. More data are warranted to confirm the current findings. Sixth, to preserve statistical power as much as possible, we also included cases' data in the analysis. Because cases do not represent the NHS population, results are not generalizable to the whole NHS cohort. Meanwhile, we excluded any participants with existing major chronic diseases at blood draw to minimize reverse causation. In addition, when we restricted the analysis within controls, similar associations were observed, suggesting that the internal validity was unlikely impaired by including cases. Lastly, although we adjusted for multiple confounders in the current analysis, we cannot fully exclude the possibility that residual confounding may still explain these findings at least partially.

Our study has several strengths. Due to the low prevalence of those with multiple low-risk lifestyle factors, a substantial sample size is required. Our study is one of the largest investigations to evaluate associations between lifestyle factors and LTL to date. The homogeneous socio-economic background of our study participants likely minimized the possibility that this factor or its correlates confound our observations [48]. Our detailed measurements of lifestyle and dietary factors enabled us to make categories that were consistent with current guidelines on lifestyle and diet and can, therefore, be translated easily into public health messages. Other strengths of the current study included the long follow-up duration and high participation rate.

### Conclusions

As the global population ages, the burden of age-related chronic disease on the economy, society, and health care systems is increasing rapidly. Numerous prior studies have demonstrated that the risk of developing diabetes, cardiovascular disease, cancer, and premature death is substantially lower among those who adhere to a healthy lifestyle pattern [11], [12], [13], [14], [15], [16], [17], [18]. Our analyses further document that a healthy lifestyle is associated with longer leukocyte telomere length, a marker of biological aging that may be a common mechanism underlying the etiology of multiple age-related diseases.

## Supporting Information

Table S1 Baseline characteristics* of the study participants in the current analysis in comparison with the rest of Nurses' Health Study participants, 1990.
(DOC)

Table S2 Telomere length z-score and relative difference by number of low-risk factors* in the Nurses' Health Study, 1990.
(DOC)

Table S3 Pair-wise association* among low-risk factors†, the Nurses' Health Study 1990.
(DOC)

Methods S1 (DOC)
