# Supplementary material for: Healthy Lifestyle and Leukocyte Telomere Length in U.S. Women
Source: PLoS One. 2012 May 31;7(5):e38374. doi: 10.1371/journal.pone.0038374 (PMC3365002; doi:10.1371/journal.pone.0038374)
Supplement: Table S1 — Baseline characteristics* of the study participants in the current analysis in comparison with the rest of Nurses' Health Study participants, 1990. (DOC) [file pone.0038374.s001.doc]

**Table S1. Baseline characteristics*** **of the study participants in the current analysis in comparison with the rest of Nurses’ Health Study participants, 1990.**

| Variables | Study participants | | NHS cohort† |
| --- | --- | --- | --- |
|  | Cases | Controls |  |
| N | 2406 | 3456 | 52884 |
| Age (year) | 58.4±0.14 | 59.0±0.11 | 56.0±0.03 |
| BMI (kg/m^2^) | 25.3±0.3 | 25.1±0.2 | 25.5±0.3 |
| Moderate to vigorous physical activity (minutes/week)‡ | 137.8±12.1 | 132.5±11.4 | 126.2±11.8 |
| Smoking status (%) |  |  |  |
| Never smoked | 41.4 | 45.5 | 44.8 |
| Past smoker | 43.0 | 40.1 | 39.2 |
| Current smoker | 15.6 | 14.5 | 16.0 |
| Alcohol intake (drink/day) | 0.4±0.04 | 0.4±0.04 | 0.4±0.04 |
| AHEI diet score | 39.6±0.5 | 39.5±0.5 | 38.6±0.5 |
| Postmenopausal status (%) |  |  |  |
| Premenopausal | 23.1 | 22.5 | 26.5 |
| Never used HRT | 20.8 | 24.9 | 29.4 |
| HT past user | 11.9 | 12.9 | 16.3 |
| HT current user | 44.3 | 39.7 | 27.8 |
| Use of aspirin (%) |  |  |  |
| <1 tablet/wk | 36.4 | 34.8 | 37.2 |
| 1-6 tablet/wk | 48.5 | 49.0 | 47.1 |
| 7+ tablet/wk | 15.2 | 16.2 | 15.7 |
| Family history of myocardial infarction (%) | 20.3 | 17.3 | 19.0 |
| Family history of diabetes (%) | 26.4 | 27.2 | 26.1 |
| Family history of cancer (%) | 17.3 | 15.6 | 14.0 |

Abbreviations: NHS, the Nurses’ Health Study; BMI, body mass index; HT, hormone therapy; AHEI, alternate healthy eating index.

*Values were age-adjusted mean±SE for continuous variables or age-adjusted proportion for categorical variables, except age *per se*.

†The same exclusion criteria were applied to the rest NHS participants. Therefore, this group was primarily consisted of participants who responded to 1990 FFQ.

‡Assessed by 1988 questionnaire. Moderate physical activities included walking at a brisk (3.0-3.9 MPH) or very brisk (≥4.0 MPH) pace. Vigorous physical activities included jogging (≥10 min/mile), running (<10 min/mile), bicycling, lap swimming, tennis, and calisthenics/aerobics/aerobic dance/rowing machine. All other variables were assessed using the 1990 questionnaire, which was administered primarily during blood collection.
