# Supplementary material for: Healthy Lifestyle and Leukocyte Telomere Length in U.S. Women
Source: PLoS One. 2012 May 31;7(5):e38374. doi: 10.1371/journal.pone.0038374 (PMC3365002; doi:10.1371/journal.pone.0038374)
Supplement: Table S2 — Telomere length z-score and relative difference by number of low-risk factors* in the Nurses' Health Study, 1990. (DOC) [file pone.0038374.s002.doc]

**Table S2. Telomere length *z*-score and relative difference by number of low-risk factors* in the Nurses’ Health Study, 1990.**

|  | Number of low-risk factors | | | | | | P for trend | |
| --- | --- | --- | --- | --- | --- | --- | --- | --- |
|  | 0  (the least healthy group) | 1 | 2 | 3 | 4 | 5  (the most healthy group) | |  |
| N (%) | 110 (1.9) | 804 (13.7) | 1592 (27.2) | 1759 (30.0) | 1198 (20.4) | 399 (6.8) | | - |
| Telomere length *z*-score (SE) | -0.195 (0.095) | -0.043 (0.035) | 0.004 (0.025) | 0.057 (0.024) | 0.008 (0.029) | 0.076 (0.050) | | 0.015 |
| % Difference (95% CI) | Reference | 16.4  (-4.0, 41.2) | 22.1  (1.2, 47.3) | 28.7  (6.6, 55.3) | 22.6  (1.4, 48.3) | 31.2  (6.6, 61.5) | | - |

Abbreviations: SE, standard error; CI, confidence interval.

*Low-risk group for each lifestyle factor was defined as non-current smoking, moderate alcohol use (1 drink/week to <2 drinks/day), a healthy body weight (18.5 kg/m2 ≤BMI< 25.0 kg/m2), exercising at moderate to vigorous intensity (≥150 minutes/week), or AHEI diet score in top two quartiles.
