# Supplementary material for: Healthy Lifestyle and Leukocyte Telomere Length in U.S. Women
Source: PLoS One. 2012 May 31;7(5):e38374. doi: 10.1371/journal.pone.0038374 (PMC3365002; doi:10.1371/journal.pone.0038374)
Supplement: Table S3 — Pair-wise association* among low-risk factors†, the Nurses' Health Study 1990. (DOC) [file pone.0038374.s003.doc]

**Table S3. Pair-wise association* among low-risk factors**†**, the Nurses’ Health Study 1990.**

|  |  | Normal BMI | | Optimal diet | | Physically active | | Moderate drinking | |
| --- | --- | --- | --- | --- | --- | --- | --- | --- | --- |
|  |  | Yes | No | Yes | No | Yes | No | Yes | No |
| Non-smoking | Yes | 2765(55.1) | 2251 (44.9) | 2661(53.1) | 2355(47.0) | 1861(37.1) | 3155(62.9) | 2341(46.7) | 2675(53.3) |
|  | No | 523(61.8) | 323(38.2) | 271(32.0) | 575(67.97) | 211(24.9) | 635(75.1) | 403(47.6) | 443(52.4) |
|  |  | P=0.0003‡ | | P<0.0001 | | P<0.0001 | | P=0.60 | |
| Normal BMI | Yes | - | - | 1742(53.0) | 1546(47.2) | 1321(40.2) | 1967(59.8) | 1643(50.0) | 1645(50.0) |
|  | No | - | - | 1190(46.2) | 1384(53.8) | 751(29.2) | 1823(70.8) | 1101(42.8) | 1473(57.2) |
|  |  | - | | P<0.0001 | | P<0.0001 | | P<0.0001 | |
| Optimal diet | Yes | - | - | - | - | 1253(42.8) | 1676(57.2) | 1386(47.3) | 1546(52.7) |
|  | No | - | - | - | - | 816(27.9) | 2114(72.2) | 1358(46.4) | 1572(53.7) |
|  |  | - | | - | | P<0.0001 | | P=0.48 | |
| Physically active | Yes | - | - | - | - | - | - | 1071(51.7) | 1001(48.3) |
|  | No | - | - | - | - | - | - | 1673(44.1) | 2117(55.9) |
|  |  | - | | - | | - | | P<0.0001 | |

*Values were N(row percent).

†Low-risk group for each lifestyle factor was defined as non-current smoking, moderate alcohol use (1 drink/week to <2 drinks/day), a healthy body weight (18.5 kg/m2 ≤BMI< 25.0 kg/m2), exercising at moderate to vigorous intensity (≥150 minutes/week), or AHEI diet score in top two quartiles.

‡All P values were based on χ^2^ test with 1 degree of freedom.
