# Supplementary material for: Healthy Lifestyle and Leukocyte Telomere Length in U.S. Women
Source: PLoS One. 2012 May 31;7(5):e38374. doi: 10.1371/journal.pone.0038374 (PMC3365002; doi:10.1371/journal.pone.0038374)
Supplement: Methods S1 — (DOC) [file pone.0038374.s004.doc]

**Methods S1**

To estimate the % difference of Y by a binary exposure variable X, we fit the following linear regression model:


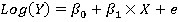
, where e is the error term.

When X=0, we will have
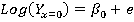
, and when X=1 we will have
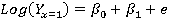
. To estimate
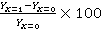
, we simply calculate
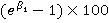
.
